# From usability to clinical impact: a systematic review of digital technologies for motor rehabilitation in multiple sclerosis

**DOI:** 10.3389/fdgth.2026.1692875

**Published:** 2026-02-16

**Authors:** Gabriele Triolo, Daniela Ivaldi, Roberta Lombardo, Angelo Quartarone, Viviana Lo Buono

**Affiliations:** Neuroimaging Laboratory, IRCCS “Neurolesi” Bonino Pulejo, Messina, Italy

**Keywords:** digital rehabilitation, digital therapeutic, motor rehabilitation, multiple sclerosis, usability

## Abstract

**Background:**

People with multiple sclerosis (PwMS) often experience motor impairments that require long-term rehabilitation. In recent years, digital technologies such as mobile applications and sensor-based systems, have been explored as tools to support motor rehabilitation in this population. However, their implementation in real-world settings critically depends on usability, which influences user engagement, adherence, and integration into clinical practice.

**Objective:**

To systematically review the usability, acceptability, and preliminary clinical impact of digital rehabilitation interventions targeting motor function in pwMS.

**Methods:**

A systematic literature search was conducted in PubMed, Scopus, Embase, and Web of Science up to June 2025, to identify studies that evaluated usability within the context of motor rehabilitation interventions delivered through digital platforms. Only studies involving pwMS were included.

**Results:**

Nine studies were included. Interventions employed Kinect-based exergaming, mobile applications, pressure-sensitive platforms, and wearable sensors. Usability was generally high, with System Usability Scale (SUS) scores >70 in several studies. Adherence ranged from moderate to high, particularly when interventions incorporated real-time feedback, personalization, and user-centered design. Preliminary improvements were observed in gait speed, balance, cognitive-motor function, and physical activity levels.

**Conclusion:**

Digital rehabilitation tools demonstrate promising usability and feasibility for motor rehabilitation in pwMS. Personalized, interactive systems designed with user-centered approaches may enhance engagement and support behavioural activation. Future large-scale trials with standardized outcomes are needed to establish clinical effectiveness and inform integration into routine care.

**Systematic Review Registration:**

https://www.crd.york.ac.uk/PROSPERO/view/CRD420251125034, PROSPERO CRD420251125034.

## 1 Introduction

**Figure 1:**
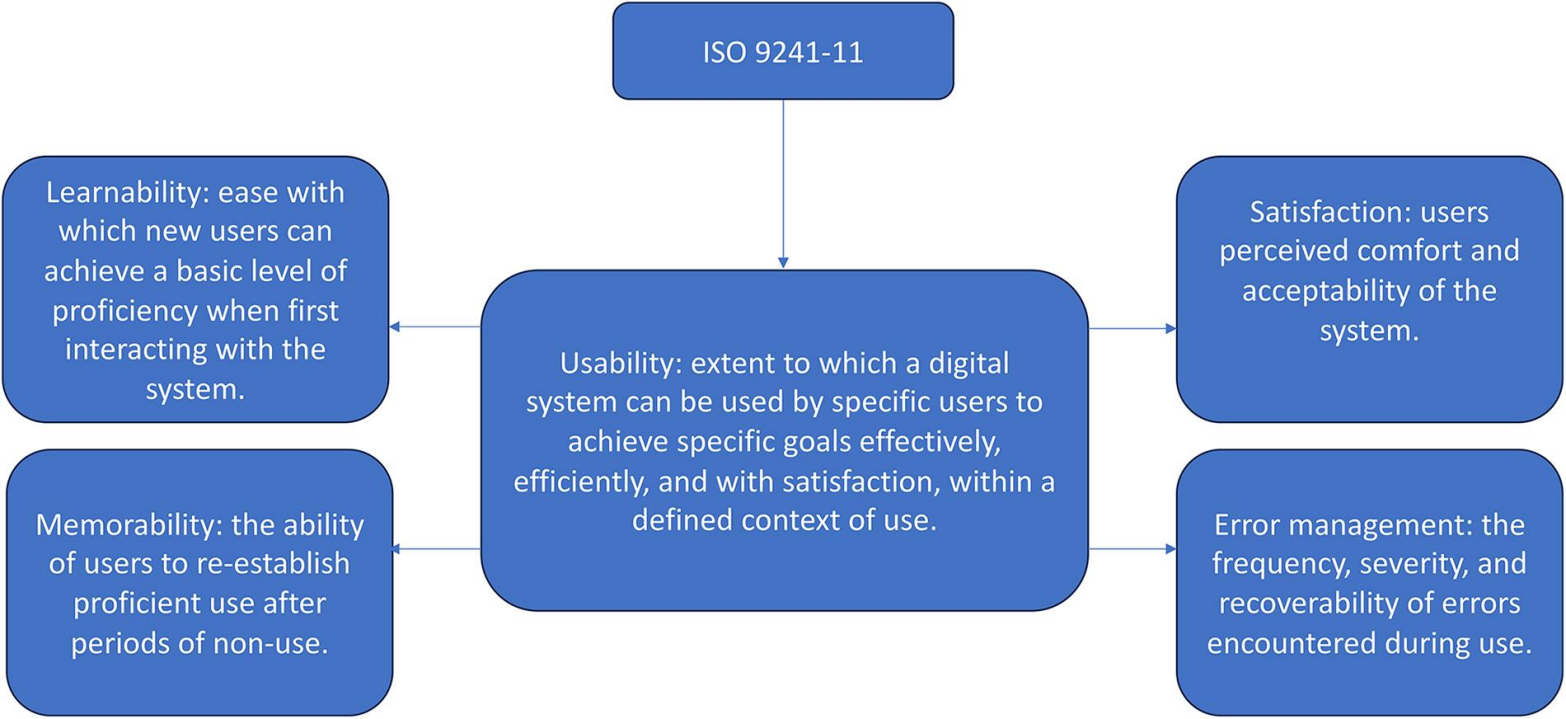
Conceptual diagram illustrating the multidimensional construct of usability.

People with multiple sclerosis (PwMS) frequently experience motor impairments, fatigue and mobility limitations that negatively affect daily functioning and quality of life (1, 2). In recent years, digital technologies, such as mobile applications, wearable sensors and interactive systems have gained growing attention as tools to support motor rehabilitation in neurological populations (3). These interventions may support the development of personalized and adaptable rehabilitation pathways, with the potential to be implemented both in clinical and home-based settings, thereby complementing conventional face-to-face therapy. Their use has been accelerated by the need to overcome access barriers and ensure continuity of care in fluctuating disease conditions (4). While the clinical potential of digital rehabilitation has been widely acknowledged, its successful implementation in real-world settings faces several challenges, including sustained user engagement, integration into clinical workflows, and continuity of use. Among these factors, usability plays a pivotal role. Defined by the ISO 9241-11 standard as the extent to which a system can be used by specific users to achieve specified goals with effectiveness, efficiency, and satisfaction in a defined context of use (5), usability is a key determinant of whether digital tools can be adopted meaningfully in clinical and everyday rehabilitation contexts. As described in Figure 1, in digital health, particularly in neurorehabilitation, usability encompasses not only these core dimensions but also additional factors such as learnability, defined as the ease with which users can begin to interact with the system, memorability, described as easily users can reestablish proficiency after a break, error management, intended as the frequency and recoverability of errors and user satisfaction (6).

These aspects are critical in populations with neurological disorders, who often face cognitive, sensory, or motor limitations that may interfere with system interaction, such as reduced proprioception or motor control, cognitive slowing or attentional deficits, and sensory processing impairments, all of which can inhibit effective, efficient, and satisfactory use of digital rehabilitation tools (7). Poor usability can lead to frustration and disengagement, ultimately diminishing the therapeutic value of the intervention and compromising its integration into long-term rehabilitation pathways, leading to a possible abandonment of the digital system (8). Tools such as the System Usability Scale (SUS) are commonly employed to quantify usability in digital rehabilitation settings, with scores above 70 typically indicating acceptable usability (9). In addition to the SUS, several other standardized tools have been employed to assess usability in digital health settings. These include the Technology Acceptance Model (TAM) scales, the Mobile App Rating Scale (MARS), and the Service User Technology Acceptability Questionnaire (SUTAQ). Furthermore, given the exploratory nature of many digital health interventions that are often at early stages of development, usability is frequently assessed through qualitative methods, including structured interviews and *ad hoc* questionnaires, which allow for a more flexible and context-sensitive evaluation of user experience.

While usability directly influences user satisfaction and adherence, its clinical relevance also lies in its capacity to support behavioural activation and functional gains. Digital tools that are perceived as easy to use and engaging are more likely to promote sustained interaction and integration into daily life, especially in the context of motor rehabilitation (10). In this regard, emerging digital technologies, including wearable sensors, smartphone applications, and exergames, offer promising means to promote physical activity in pwMS (11). Wearable sensor systems can remotely and objectively assess mobility metrics like walking speed, gait stability, and daily step counts, serving as both monitoring and motivational tools to encourage movement in free-living conditions (12). Among the various therapeutic targets in multiple sclerosis (MS), promoting regular physical activity represents a crucial goal, as inactivity and sedentary behaviour are highly prevalent and contribute to the progression of motor disability. A recent meta-analysis of randomized controlled trials documented that exercise interventions, whether aerobic, resistance, or multicomponent training, produce significant improvements in balance, walking ability and endurance, fatigue reduction, and overall quality of life in pwMS (13). The physiological underpinnings include meaningful enhancements in neurotrophic support, immune modulation, and neuroplasticity, actors contributing to both central nervous system repair and functional gains (14). Additionally, engagement in physical activity is indirectly associated with better quality of life through psychological and behavioural mediators such as reduced fatigue and depression, enhanced self-efficacy, pain relief, and increased social support (15).

Given the growing interest in digital solutions for motor rehabilitation in MS, and the crucial role of usability in determining their real-world applicability, this review aims to synthesize current evidence on the usability, acceptability, and preliminary clinical impact of digital rehabilitation interventions in pwMS. The objective is to explore how usability and user experience have been evaluated in digital motor rehabilitation programmes for pwMS and to investigate whether these aspects may be associated with more favourable clinical and motor outcomes. By mapping and critically appraising the existing literature, this review seeks to identify both the opportunities and limitations associated with the integration of such technologies into rehabilitation pathways. In particular, it examines how these digital tools have been applied in practice and what evidence exists regarding their usability and clinical promise.

## 2 Methods

This systematic review was conducted to investigate the usability, acceptability, and preliminary clinical impact of digital rehabilitation interventions in pwMS. The review was conducted in accordance with the Preferred Reporting Items for Systematic Reviews and Meta-Analyses (PRISMA) guidelines (16). In addition, the protocol for this systematic review has been published and registered with the International Prospective Register of Systematic Reviews (PROSPERO, CRD420251125034).

The research question was structured according to the PICO framework (Population, Intervention, Comparison, Outcome) to ensure clarity and methodological rigor (17). The population of interest was pwMS. The interventions considered were rehabilitation programs delivered through digital health technologies, such as mobile applications, exergames, wearable sensor-based systems. Studies were eligible regardless of the presence of a comparison group. The primary outcomes of interest were usability, acceptability, and user engagement with the digital interventions, while secondary outcomes included preliminary effects on clinical parameters such as gait, balance and physical activity.

A systematic search was conducted for all peer-reviewed articles published up to June 2025, using the following databases: PubMed, Scopus, Embase, and Web of Science. The search was performed between April and June 2025. The search combined the following terms in PubMed:

- (“Multiple Sclerosis"[Mesh] OR “multiple sclerosis” OR “MS”) AND (“smartphone” OR “mobile application” OR “mobile app” OR “tablet” OR “mHealth” OR “mobile health” OR “digital health” OR “digital therapeutics” OR “Serious Games”) AND (“rehabilitation” OR “motor recovery” OR “physical therapy” OR “physical activity” OR “gait” OR “walking” OR “balance” OR “mobility”) AND (“adherence” OR “compliance” OR “engagement” OR “feasibility” OR “usability” OR “acceptability” OR “user experience” OR “satisfaction” OR “retention”)

The complete search strings used for each database are available in Supplementary Appendix A.

Titles and abstracts retrieved from the searches were screened for eligibility by two independent reviewers (G.T. and D.I.). Subsequently, full-text articles were assessed by one reviewer (G.T.) to determine whether they met the predefined inclusion criteria. Any discrepancies between reviewers were resolved through discussion, and, when necessary, with the involvement of a third reviewer (V.L.B.).

Data from the included studies were extracted using a standardized Excel spreadsheet specifically designed for the review. One reviewer (G.T.) independently extracted data from each study, and all entries were subsequently cross-checked by a second reviewer (R.L.) to ensure accuracy and consistency. Disagreements were resolved through discussion among the reviewers.

Studies were eligible for inclusion if they met the following criteria:

- People diagnosed with MS, of any age or sex.
- Digital rehabilitation interventions delivered through digital devices for mobile use or clinical settings (e.g., smartphone applications, wearable sensors, motion-tracking systems, exergames, tablet-based tools, or remote physiotherapy platforms).
- The usability of the platform was systematically evaluated using established assessment methods or *ad hoc* questionnaire.

Exclusion criteria were:

- Studies focusing exclusively on symptom monitoring or diagnostic assessment without a rehabilitative aim.
- Studies involving populations other than pwMS.
- Protocols, reviews, conference abstracts, and purely qualitative studies lacking quantitative or mixed-method data related to adherence, usability or motor outcomes were excluded.
- Studies not reporting any outcome measures related to physical, usability or acceptability outcome.

### 2.1 Risk of bias assessment

The assessment of risk of bias for the included studies was conducted independently by two reviewers (G.T. and D.I.). Discrepancies encountered at this stage, as well as during prior phases of the review, were resolved through discussion with a third reviewer (R.L.), who provided the final decision. For the randomized controlled trials included in this review the Rob 2 tool was used (18). RoB 2 evaluates the following five domains: (I) bias arising from the randomization process, (II) bias due to deviations from intended interventions, (III) bias due to missing outcome data, (IV) bias in measurement of the outcome, (V) bias in selection of the reported result. Each domain is judged as low risk, some concerns, or high risk, and contributes to an overall judgment of the risk of bias. For all other included studies, the ROBINS-I tool was used (19). ROBINS-I evaluates the following seven domains: (I) bias due to confounding, (II) bias in selection of participants into the study, (III) bias in classification of interventions, (IV) bias due to deviations from intended interventions, (V) bias due to missing data, (VI) bias in measurement of outcomes (VII) bias in selection of the reported result. Each domain is rated as low, moderate, serious, critical risk of bias, or no information, and the overall risk of bias is derived accordingly. To graphically represent the results obtained using the tools, a table was finally produced using the Robvis tool (20).

## 3 Results

**Figure 2:**
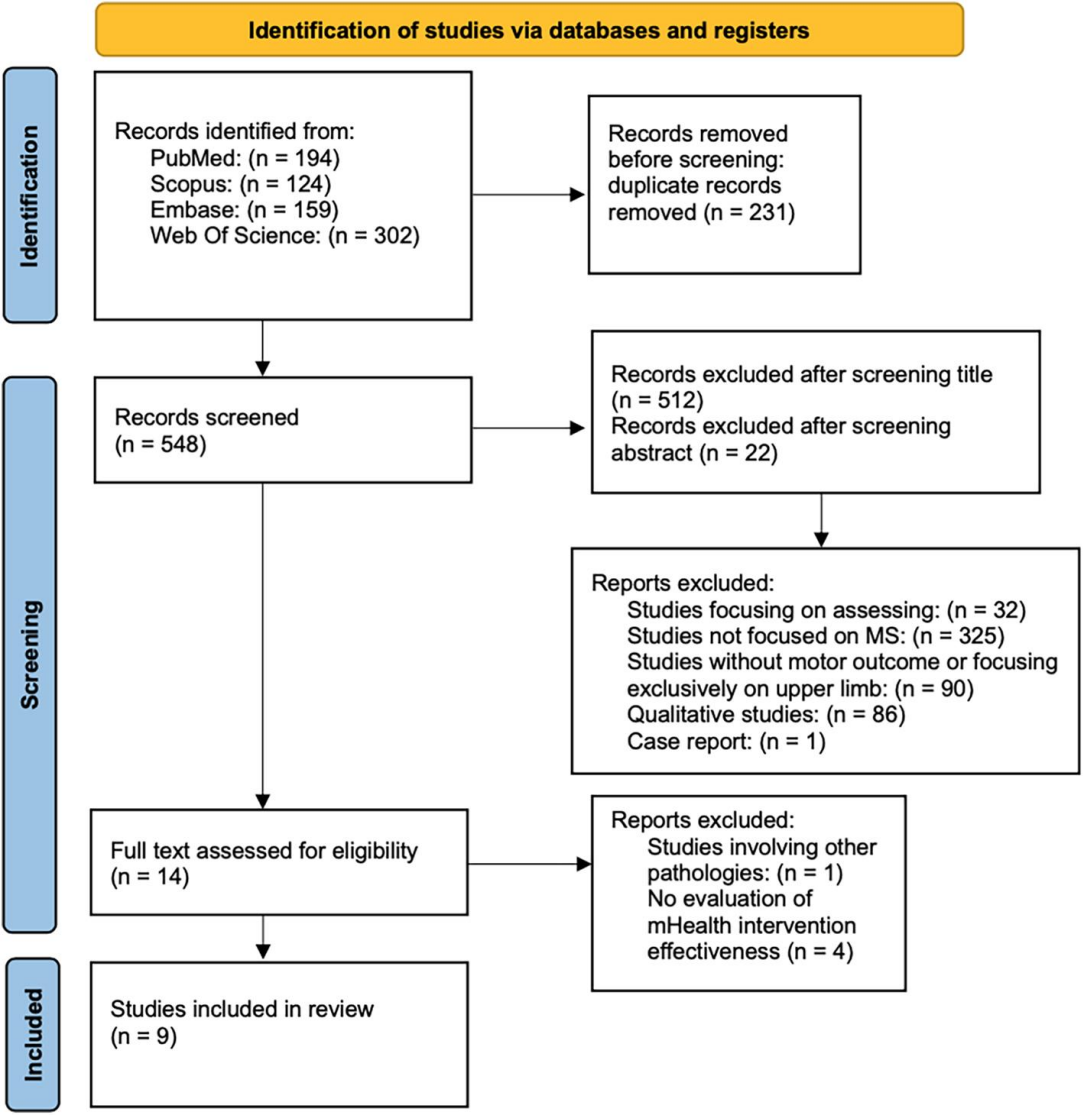
PRISMA flow diagram of the studies selection progress.

A total of 779 records were identified through database searching. After removing duplicates, a total of 548 records were screened based on title, 36 articles were further assessed by abstract, and 14 full-text articles were retrieved for detailed evaluation. Following the full-text screening, 9 studies met the inclusion criteria and were included in the final synthesis. All full-text articles were independently assessed for eligibility by two reviewers (G.T. and D.I.). The complete study selection process is illustrated in the PRISMA flow diagram (Figure 2).

Table 1 summarizes the main characteristics of the studies included in this review, detailing key elements such as study design, participant characteristics, intervention duration, type of digital device employed, specific objectives, and main findings. Across the included studies, sample sizes ranged from 9 to 38, and the intervention encompassed a variety of digital rehabilitation modalities, including Kinect-based exergaming systems, pressure-sensitive platforms, wearable sensors, and mobile applications. Interventions were conducted in both supervised and home-based settings, with durations ranging from single sessions to 12-week programs. Across studies, outcomes focused on usability, acceptability, and preliminary clinical effects, with interventions targeting motor performance, physical activity, balance and cognitive-motor interference (Table 1).

**Table 1:** Main characteristics of included studies.

Authors and year	Study design	Population	Duration	Device	Objective	Intervention	Principal findings
Chanpimol et al., (2020) (21)	Single-arm pre-post pilot study	10 pwMS	12 weeks	Xbox Kinect sensor, Dell Latitude tablet	To evaluate acceptability and physical effects of an exergaming intervention.	The physical therapist remotely monitored and adjusted the exergames based on system-generated reports and participant feedback.	The intervention was acceptable and may improve gait and mobility.
Tacchino et al., (2023) (22)	Development and usability study	9 pwMS	3 test session	Microsoft Kinect V2, Intel Next Unit computer	To describe the design, development, and usability testing of MS-FIT, a Kinect-based tool implementing Pilates exercises customized for pwMS.	Gamified virtual journey with avatar-based Pilates training; real-time feedback via motion tracking, directional cues, and rewards No synchronous supervision by a physical therapist is provided.	All participants completed the sessions and reported the tool easy to use, playable, enjoyable and satisfactory.
Schättin et al., (2021) (23)	Mixed methods study	16 pwMS during the first study, 25 pwMS during the second study	Single session during the first study, 4 weeks for the second study	Dividat Senso plate	To develop and evaluate effective and attractive exergames that combine training principles with game design elements, in order to address MS-specific deficits and improve patients’ quality of life.	Two consequent studies: in the first study a single session was conducted. The second study was conducted after redesigning the exergame.	User-centred exergames are a promising and well-accepted training approach for MS patients, with the potential to improve physical and cognitive function, brain-body communication, and quality of life, and to reduce the risk of falls and social isolation.
Tacchino et al., (2020) (24)	Single-arm pilot study	15 pwMS	8 weeks	CMI-APP, Android tablet	To describe the design and development of CMI-APP, and to present the preliminary results of the study.	Dual-task training with tablet-based app targeting cognitive-motor interference.	The intervention was safe, highly usable, motivating, and well accepted for dual-task training in pwMS.
Nasseri et al., (2020) (25)	Pilot RCT	38 pwMS	3 months	Samsung s-4 mini, smartphone app	To investigate the feasibility of a smartphone application to improve physical activity in people with chronic progressive multiple sclerosis.	The intervention group received access to a smartphone application that provided EBPI, educational videos and illustrations, as well as simplified accelerometer-based feedback on physical activity. The control group received a two-page leaflet with general information on the health benefits of exercise, without EBPI content or personalized feedback.	No significant differences in physical activity levels were observed between groups. However, participants in the intervention group reported high levels of app acceptability and showed a trend toward increased motivation for engaging in a more active lifestyle.
Stephens et al., (2022) (26)	Pilot feasibility study	15 youth pwMS	12 weeks	Fitbit Charge 2	To determine the feasibility of a physical activity promotion program in youth with MS.	The app was developed based on feedback from adolescents with MS and integrates MS-specific content. It is grounded in SCT, incorporating key constructs such as self-efficacy, outcome expectations, goal setting, perceived barriers, and social support.	ATOMIC program was found to be feasible and well accepted by participants. No significant changes were observed in MVPA, cardiorespiratory fitness, fatigue, or depression.
Van Geel et al., (2020) (27)	Pilot feasibility study	19 pwMS	10 weeks	Participant personal smartphone	To evaluate the feasibility of prolonged use of the WalkWithMe, a personalized mobile application that supports pwMS in walking at home, and its effect on physical activity, walking, fatigue and cognition in persons with MS.	WalkWithMe allows users to track their walking activities and follow up on their progress. Participants were instructed to use the app in their community setting.	WalkWithMe was successful at improving categories of self-reported physical activity, lower limb functional strength, hand function and cognition
Lozano-Quilis et al., (2014) (28)	RCT	11 pwMS	10 weeks	Microsoft Kinect	To evaluate the effectiveness of RemoviEM, a system based on virtual reality and natural user interfaces for performing motor rehabilitation exercises.	The system was developed to perform motor exercises in a motivating and intuitive way. It consists of three main exercises: TouchBall, TakeBall, and StepBall, which are primarily useful for improving balance and load transfers.	RemoviEM is an effective and engaging Kinect-based rehabilitation system for pwMS. It significantly improved balance outcomes compared to standard rehabilitation, with high levels of usability and user satisfaction.
Meldrum et al. (2024) (29)	Usability and feasibility pilot study	16 pwMS	Mean 12 weeks (±2.2)	Vertigenius™, smartphone application, software for the clinician	To investigate the usability of a personalized digital vestibular rehabilitation system.	Vestibular physical therapy was delivered through a personalized digital system. The sensor provided real-time feedback on the correct frequency of head movement compared to the prescription and transmitted the information to the portal. The app provided video guidance for each exercise.	This digital system is acceptable, usable, and promising for improving symptoms, balance, and gait in pwMS, while also offering a valuable approach for remote care delivery.

### 3.1 Sensor

Several studies employed sensor-based technologies for motor rehabilitation in pwMS. The following subsections summarize the usability findings, adherence patterns, and clinical outcomes reported for each sensor type.

#### 3.1.1 Kinect-based sensor

Three studies employed Kinect-based systems to deliver motor rehabilitation interventions in pwMS (21, 22, 28). RemoviEM was tested in an RCT, showing significant improvements in balance performance compared to conventional therapy. The intervention consisted of three motor tasks focusing on trunk control, weight shifting, and coordination. Compared to the control group, participants using RemoviEM showed statistically significant improvements in balance performance assessed with the Berg Balance Scale (BBS) and Single Leg Balance test. Additionally, participants expressed high enjoyment and reported no adverse effects (28). An exploratory study evaluated the feasibility and preliminary clinical effectiveness of a 12-week individualized telerehabilitation program using a software integrated with the Xbox Kinect sensor. The intervention, delivered remotely through clinical video teleconferencing, focused on balance, strengthening, and functional exercises tailored to each patient. PwMS demonstrated significant improvements in ambulation speed (25-Foot Walk Test), walking endurance (2-Minute Walk Test), and overall lower extremity physical function (Short Physical Performance Battery). Participants reported high satisfaction with the intervention and no adverse events were noted (21). A study introduced a Kinect-based intervention that integrated pilates exercises specifically adapted for pwMS, developed through a user-centred design approach involving both healthcare professionals and patients. Preliminary usability testing demonstrated high ratings in ease of use, playability, and acceptance, suggesting good feasibility for independent use in outpatient settings. Through a user-centred design approach involving both healthcare professionals and patients, the authors performed usability tests with nine pwMS across three outpatient sessions. The system was rated highly regarding ease of use, playability, and acceptance, suggesting good feasibility and potential to enhance patient engagement, although no clinical outcomes were assessed in this study (22).

#### 3.1.2 Wearable sensor

Another study evaluated the usability and outcomes of a remotely delivered vestibular physical therapy intervention for pwMS, employing a wearable inertial head sensor combined with a smartphone application. The system enabled real-time biofeedback and remote monitoring during gaze stabilization exercises prescribed to address dizziness, balance deficits, and gait disturbances commonly observed in pwMS. Sixteen participants were recruited, of which twelve completed the program, engaging in a mean of 12 weeks of treatment. Results indicated excellent system usability, with participants particularly valuing the convenience, accessibility, and immediate performance feedback provided by the digital setup. Statistically significant improvements were observed in dynamic visual acuity (*p* = 0.004), balance (Mini-BESTest, *p* = 0.004), and dynamic gait function (Modified Dynamic Gait Index, *p* = 0.008). Participants also demonstrated significant increases in head movement frequencies during gaze stabilization exercises, concurrent with reductions in dizziness intensity, suggesting improved vestibular function and tolerance to head movements. These findings support the use of wearable sensor technology as promising tool for delivering remote vestibular rehabilitation in pwMS, offering a feasible solution to overcome common barriers such as travel demands, symptom provocation, and limited supervision during home-based therapy (29).

#### 3.1.3 Pressure-sensitive sensor

A study evaluated the feasibility and usability of a customized exergaming intervention specifically developed for pwMS, employing Dividat Senso, a pressure-sensitive platform that captures lower-limb movements, weight shifts, and stepping responses. Through a user-centred design approach involving focus groups with therapists and pwMS, the authors developed three interactive exergames targeting motor functions such as static and dynamic balance, coordination, and cognitive domains including attention, visuospatial skills, and executive functions. Twelve pwMS participated in four supervised training sessions over a period of four weeks, during which individual difficulty levels were adjusted to provide tailored cognitive and physical challenges. Results from quantitative usability analyses indicated good to excellent user acceptance. Participants reported substantial enjoyment, high motivation, increased self-confidence, and a positive shift in focus from their limitations to their capabilities. Despite no clinical outcomes being assessed, qualitative feedback emphasized the potential of this system to enhance patient engagement and adherence to therapy by providing an enjoyable and motivating rehabilitation experience. The results point to the utility of participatory design approaches in creating engaging rehabilitation tools and support the integration of sensor-based platforms into broader MS rehabilitation strategies (23)..

Is important to notice that none of the included studies reported formal clinical validation data for the sensors used.

### 3.2 Mobile app tools

Four studies explored the feasibility, usability, and preliminary efficacy of mobile apps as tools to enhance physical activity and cognitive-motor performance in pwMS (24–27).

#### 3.2.1 Promoting physical activity app

A community-based feasibility study investigated the effects of WalkWithMe, a smartphone application designed to encourage walking in pwMS through personalized goal setting and real-time feedback. Over a 10-week period, twelve participants completed the program, reporting significant gains in leisure-related activity, functional strength, manual dexterity, and aspects of cognitive function. While objective measures of walking performance did not show clinically relevant improvements, user feedback indicated high levels of satisfaction and engagement with the digital platform (27). The feasibility of a smartphone application designed to promote physical activity in pwMS was assessed in a three-month randomized controlled pilot trial. The intervention delivered evidence-based multimedia content and basic feedback on activity levels. Thirty-eight participants were randomized to either the app-based intervention or a control group receiving standard informational material. Although usability ratings were positive, no significant between-group differences were observed in physical activity levels. However, users of the app reported increased motivation to remain active, indicating that incorporating more interactive and personalized elements may enhance future effectiveness (25).

The ATOMIC mobile application, a theory-driven intervention designed to promote physical activity in adolescents with MS, was evaluated over a 12-week period to assess its feasibility and preliminary effects. The program incorporated personalized coaching, goal-setting, educational content, and social support elements based on Social Cognitive Theory. Fifteen participants completed the intervention, demonstrating high adherence to the app features and good compliance with wearable activity monitors. Although no significant changes were observed in moderate-to-vigorous physical activity, an average increase of 8.4% in daily step counts was recorded. Participants reported enhanced social support from peers and family, while a decrease in outcome expectations suggested the need to strengthen motivational components in future app iterations (26).

#### 3.2.2 Motor-cognitive dual-task exercises app

A tablet-based intervention (CMI-APP) was developed to assess and train cognitive-motor interference (CMI) in pwMS through dual-task exercises combining cognitive and motor components. The app was evaluated in a multicenter pilot study involving 15 participants who completed 20 training sessions. Participants rated the training as moderately challenging and enjoyable, supporting good usability and acceptability. Although no clinical outcomes were measured, the positive user feedback suggested promising feasibility for future implementation (24).

Evidence from the reviewed studies underscores the potential of mobile applications as versatile digital health interventions, incorporating elements such as personalized coaching, goal setting, real-time feedback, and self-monitoring to facilitate physical activity and cognitive-motor engagement in pwMS.

### 3.3 Intervention setting, delivery mode, and dosage characteristics

The included studies varied substantially in terms of intervention setting, delivery mode, and duration. Most mobile app and sensor-based interventions were conducted in unsupervised settings, allowing participants to engage with the technology independently or with minimal remote support. Specifically, interventions such as WalkWithMe, ATOMIC, VertiGenius were deployed for autonomous use in daily environments (26, 27, 29), Kinect-based studies were conducted either in supervised outpatient settings (22, 28), or in hybrid telerehabilitation formats combining remote monitoring and clinician-guided progression via video teleconferencing (21). Intervention duration ranged from single-session protocols to 3 months programs, with a common range between 8 and 12 weeks. Training frequency was often flexible or progressive, typically involving 2–5 sessions per week, with adjustments based on individual goals and tolerance. Some interventions predefined a fixed number of sessions (20 dual-task trainings), while others offered self-paced engagement (24). This heterogeneity reflects both the exploratory nature of the interventions and the adaptability of digital tools to different rehabilitation contexts.

### 3.4 Usability and user-experience

Across the included studies, usability and user experience were evaluated using heterogeneous approaches. Table 2 provides an overview of the outcome measures used across the included studies, distinguishing between those assessing usability, acceptability, adherence and engagement, and those evaluating physical performance or clinical parameters. Usability was typically assessed through validated tools such as the SUS, *ad hoc* questionnaires, or structured interviews, while engagement and motivation were explored through adherence rates or intrinsic motivation inventories. Adherence and engagement were reported in 5/9 studies using various metrics (e.g., dose adherence/session completion, wearable wear-time) while usability was mostly evaluated using quantitative questionnaires, with only a subset of studies also incorporating supplementary qualitative inputs. Most investigations relied on study-specific questionnaires or satisfaction surveys, whereas validated instruments were used less frequently (SUS 2/9, MARS 1/9, SUTAQ 1/9). Physical outcome measures varied according to the intervention target and included standardized assessments of gait (e.g., 6 Minute Walk Test, Timed 25 Foot Walk), balance (e.g., BBS, Mini-BESTest), strength (e.g., 5-Sit To Stand), manual dexterity (e.g., Nine Hole Peg Test), and cognitive functioning (e.g., Symbol Digit Modality Test). This heterogeneity reflects the diverse goals and technologies underpinning the digital rehabilitation interventions analyzed in the review (Table 2).

**Table 2:** Main measures used.

Authors and year	Usability measures	Adherence/engagement measures	Physical measures
Chanpimol et al., (2020) (21)	Satisfaction questionnaire (90% very satisfied; 10% satisfied; 100% would use again; 100% would recommend; 90% reported cost savings); post-intervention questionnaire (60% reported several cost savings).	Exercise sessions/week: 2.5 ± 0.8; total sessions: 32.4 ± 10.6; weeks with ≥3 sessions: 7.0 ± 3.3; overall adherence: 58.3%; dose adherence: 83%.	SPPB, 25FW, MFIS, MSWS-12, 2MWT.
Tacchino et al., (2023) (22)	8 items questionnaire *ad hoc* measuring ease of use, playability, enjoyment, satisfaction and acceptance, average item scores ranged from 3.78–4.33 (on a 1–5 scale).	n.a.	n.a.
Schättin et al., (2021) (23)	SUS (89.7 pre, 82.5 post), FSS (overall flow 5.9 pre, 5.8 post), game flow questionnaire (5.0 pre, 5.1 post).	Ad hoc questionnaire (High enjoyment, good control, immersive experience, no adverse events).	BORG motor, ABC, MSIS.
Tacchino et al., (2020) (24)	IMI (motivation): high perceived value and enjoyment, moderate effort, low pressure.	Percentage of performed sessions (91%).	n.a.
Nasseri et al., (2020) (25)	Ad hoc questionnaire focusing on comprehensibility, usability, content quality (mean ratings 3.7/5).	n.a.	6MWT, TTW, 5-STS, MSFC, T25FW, NHPT, SDMT, Actigraph.
Stephens et al., (2022) (26)	inferred from adherence and engagement:	Fitbit wear time (89%), coaching calls (80%), text interactions (61 ± 36 messages over 12 weeks).	Social cognitive scale, Actigraph (GT3X)
Van Geel et al., (2020) (27)	Semi-structured interview, MARS.	n.a.	6MWT, T25FW, MSWS-12, 5-STS, 9HPT.
Lozano-Quilis et al., (2014) (28)	SEQ (55.56/65 (SD = 5.94), direct qualitative feedback from patients and clinical observations.	n.a.	BBS, Tinetti Balance Scale, SLB, 10MWT, TUG, Anterior Reach Test in standing position.
Meldrum et al., (2024) (29)	SUS (81 ± 14), SUTAQ (enhanced care: 5/6, increased access: 4.9/6, satisfaction 5.5/6), PEI (5.8/12).	Digitally measured adherence to vestibular exercises: mean 60% ± 18.4.	Mini-BESTest, mDGI, GS, DVA, mCTSIB, Head Kinematics.

SPPB, short physical performance battery; 25FW, 25-foot walk; MFIS, Modified Fatigue Impact Scale; MSWS-12, multiple sclerosis walking scale-12; 2MWT, 2-minute walk test; SUS, System Usability Scale; FSS, Flow Short Scale; ABC, Activities-specific Balance Confidence; MSIS, Multiple Sclerosis Impact Scale; IMI, Intrinsic Motivation Inventory; 6MWT, 6 min walking test; TTW, timed tandem walking; 5-STS, Five Times Sit To Stand test; MSFC, Multiple Sclerosis functional composite; T25FW, timed 25 foot walk; NHPT, nine hole peg test; SDMT, Symbol Digit Modality Test; MARS, Mobile App Rating Scale; SEQ, Suitability Evaluation Questionnaire; BBS, Berg Balance Scale; SLB, Single Leg Balance test; 10MWT, 10-meter Walking Test; TUG, Time “Up and Go” Test; SUTAQ, Service User Technology Acceptability Questionnaire; PEI, Patient Enablement Instrument; mDGI, modified Dynamic Gait Index; GS, Gait Speed; DVA, Dynamic Visual Acuity; Modified, mCTSIB, Modified Clinical Test of Sensory Interaction in Balance.

The SUS was used in studies involving Dividat Senso and VertiGenius, with scores exceeding 80/100, suggesting high perceived usability (23, 29). In the vestibular rehabilitation program, usability assessment was complemented by the Service User Technology Acceptability Questionnaire (SUTAQ), which confirmed user satisfaction and confidence in remote care delivery (29). The Mobile App Rating Scale (MARS) was used in the evaluation of WalkWithMe, capturing multiple dimensions of perceived app quality, including engagement and functionality (27). Other studies adopted qualitative or semi-structured approaches. The CMI-APP intervention reported high adherence (91%) and used the Intrinsic Motivation Inventory alongside a structured usage log, providing insight into perceived challenge and enjoyment (24). In ATOMIC, usability was inferred from sustained engagement with app features and compliance with wearable activity trackers (26). Similarly, another intervention relied on an *ad hoc* questionnaire to assess user experience, revealing positive perceptions despite the absence of behavioural impact (25). The MS Fitness Intervention was evaluated through an 8-item usability questionnaire specifically developed to measure ease of use, playability, satisfaction, and perceived acceptance (22). In RemoviEM, a Kinect-based system, usability data were collected through a suitability evaluation questionnaire, direct patient feedback, and clinical observation (28). Finally, in a remotely supervised exergame program, user experience was evaluated through a satisfaction survey and a post-intervention questionnaire, which indicated positive acceptance and engagement, while adherence was moderate (58.3%) (21). Despite methodological heterogeneity, high usability and acceptability were consistently reported across studies. Features such as intuitive interfaces, real-time feedback, gamified environments, and personalization were frequently cited as facilitating factors. Importantly, several interventions incorporated a user-centred or participatory design approach, involving patients and clinicians in iterative development cycles. This was especially evident in the development of Dividat Senso, the Pilates-based Kinect intervention, and the ATOMIC app, where co-design processes contributed to improved alignment between technological functionalities and user needs (22, 23, 26). Collectively, these findings reinforce the idea that usability is not only essential for initial adoption, but also a key driver of engagement, adherence, and long-term sustainability in digital rehabilitation for pwMS.

### 3.5 User-centred design in digital rehabilitation development

Several interventions included in this review were developed following a user-centred design which emphasizes the active involvement of end-users. This methodology was particularly evident in the development of gamified systems and mobile applications. Dividat Senso was designed through iterative consultations with pwMS and rehabilitation professionals, ensuring that training contents and difficulty levels addressed specific functional and cognitive needs (23). Similarly, a Kinect-based intervention incorporating Pilates exercises was developed with direct input from both patients and clinicians, resulting in a system rated as highly usable and engaging (22). In the case of adolescent-focused interventions, such as the ATOMIC app, focus groups and interviews with young users guided the creation of age-appropriate content, social support components, and behavioural strategies grounded in Social Cognitive Theory (26). These participatory processes enhanced not only usability and acceptability, but also the ecological validity and relevance of the interventions in everyday life. Overall, the adoption of user-centred design principles contributed to more intuitive interfaces, improved alignment with user expectations, and greater potential for long-term engagement. These findings support the integration of co-design methodologies as a foundational element in the development of digital rehabilitation tools for pwMS.

### 3.6 Risk of bias

**Figure 3:**
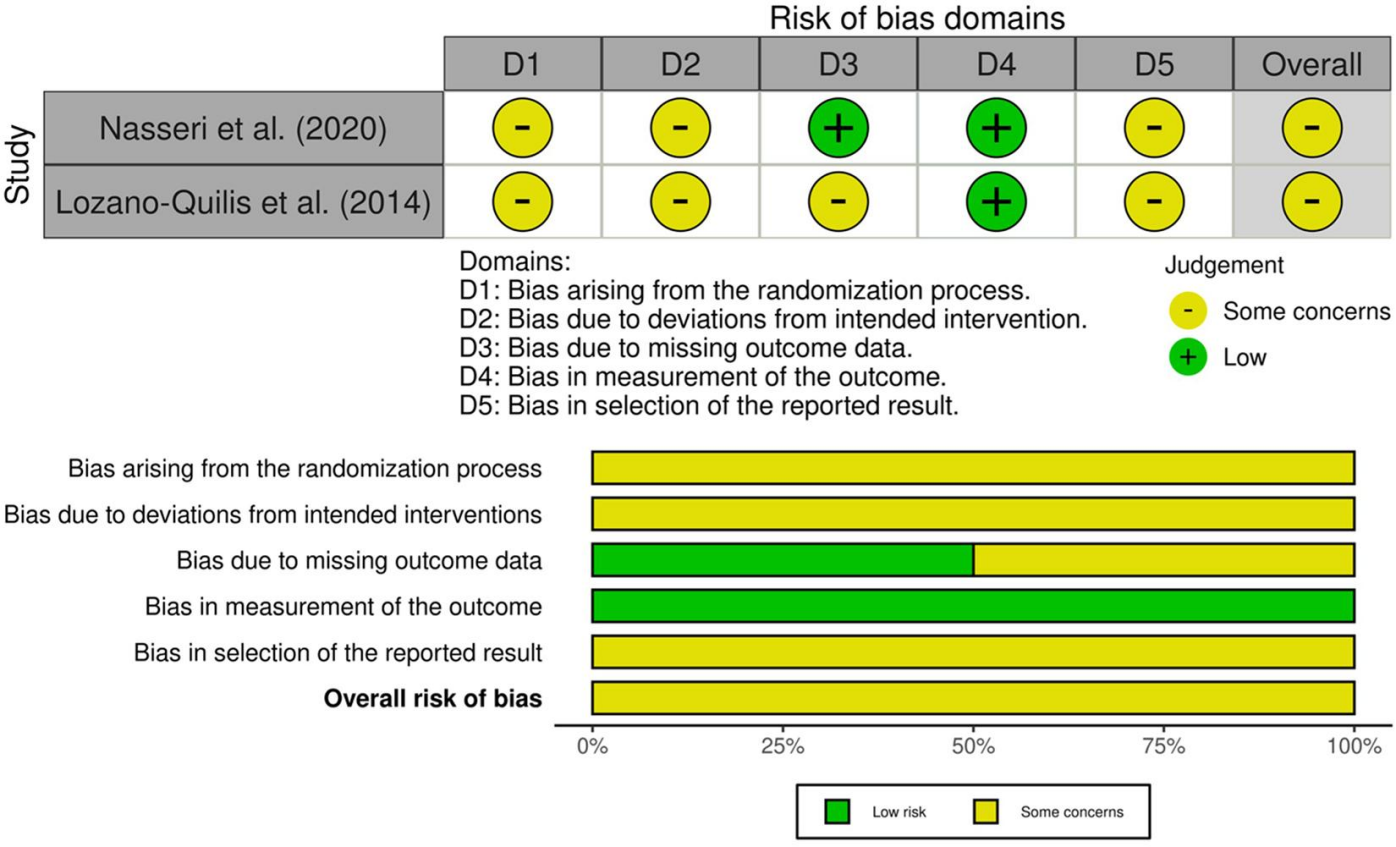
Graphical representation of the risk of bias assessment performed using the revised cochrane risk of bias 2.0 tool (RoB 2), summarizing the judgments across the five domains for each included randomized controlled trial.

The methodological quality of the included randomized controlled trials was assessed using the revised Cochrane Risk of Bias (RoB 2.0) tool (18). Figure 3 provides a graphical representation of the risk of bias assessment. A study involving a smartphone-based intervention (25), was rated as having “some concerns” overall, due to baseline differences between groups, potential deviations resulting from participants' awareness of the assigned interventions, and the absence of a pre-specified analysis plan. Specifically, bias arising from the randomization process (D1), deviations from intended interventions (D2), and selection of the reported results (D5) were each judged as “some concerns,” whereas the measurement of outcomes (D4) and handling of missing data (D3) were assessed as “low risk”. Similarly, a study evaluating a Kinect-based virtual reality intervention (28) was rated as having “some concerns” overall, primarily due to inadequate information regarding allocation concealment, the absence of an intention-to-treat analysis, unexplained missing outcome data, and the lack of a pre-specified analysis plan. Specifically, the randomization process (D1), deviations from intended interventions (D2), missing outcome data (D3), and selection of the reported results (D5) were each judged as “some concerns”, whereas bias in the measurement of outcomes was classified as “low risk” (D4). These methodological limitations should be considered when interpreting the findings and assessing their applicability to clinical practice, real-world applicability should be interpreted cautiously until confirmed in adequate trials.

**Figure 4:**
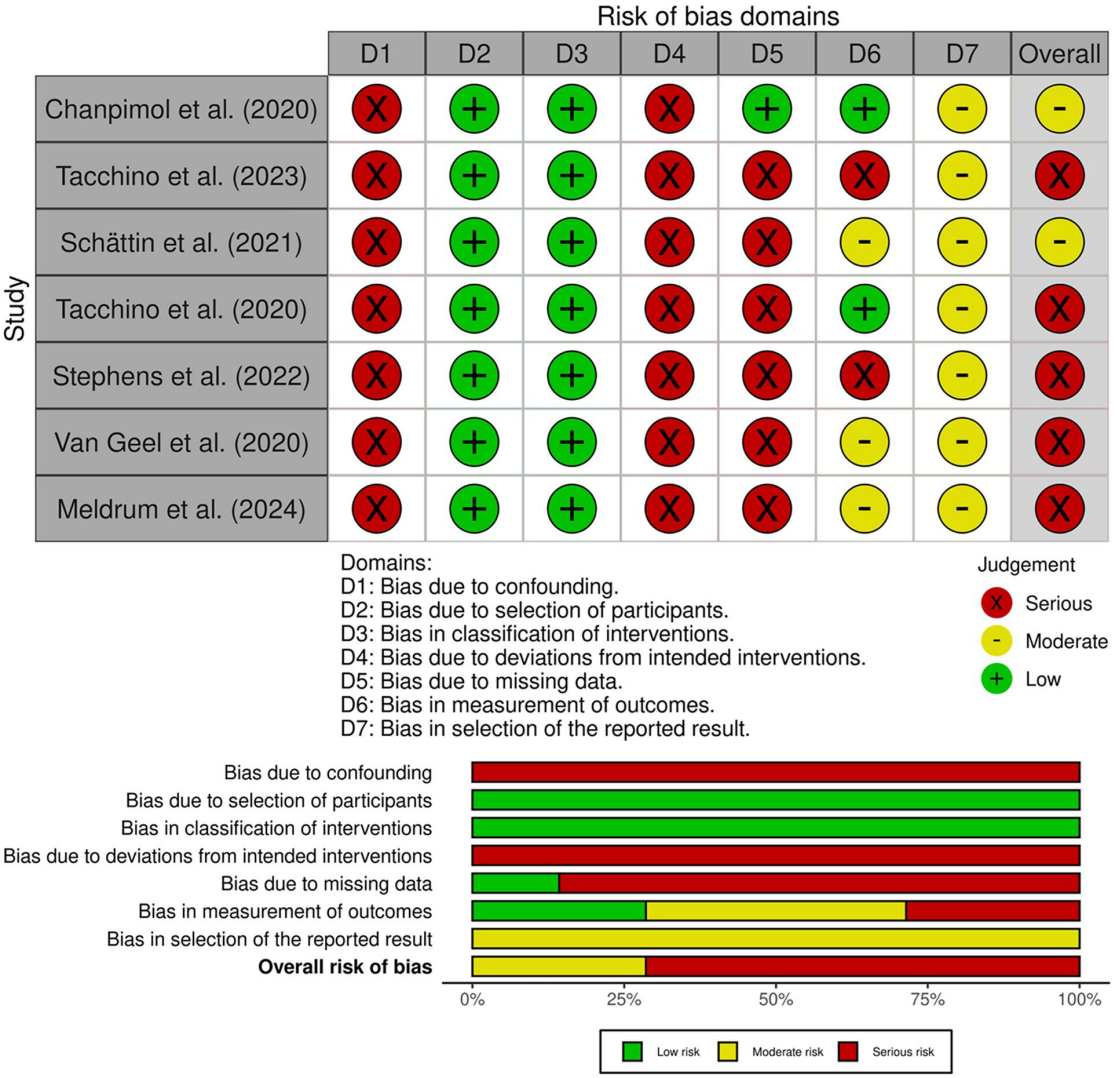
Risk of bias assessment for non-randomized studies using the ROBINS-I tool, reporting domain-level judgments and overall risk.

The risk of bias across the included studies was evaluated using the ROBINS-I tool (19). As reported in Figure 4, most studies were judged to be at serious overall risk of bias, primarily due to limitations in the control of confounding, handling of missing data, and analysis of deviations from intended interventions. All studies were rated as having a serious risk of bias due to confounding (D1), given the lack of statistical adjustment for key baseline characteristics. Bias due to classification of the intervention (D2) and selection of participants (D3) was low across the studies, as participants were clearly and consistently assigned to the intervention group, and recruitment procedures did not rely on post-baseline information. Bias due to deviations from intended interventions (D4) was judged to be serious in all studies. This was mainly attributable to the use of complete-case analyses restricted to participants who completed the intervention, without any statistical methods to account for protocol deviations (21–24, 26, 27, 29). Similarly, bias due to missing data (D5) was rated as serious in most studies (22–24, 26, 27, 29). Regarding bias in measurement of outcomes (D6), two studies were rated as having a serious risk of bias. In both cases, usability was assessed through non-standardized questions or qualitative feedback, without the use of validated instruments. The self-reported nature of the data, combined with the absence of blinding and structured assessment protocols, contributed to a high risk of bias in outcome measurement (22, 26). Three studies were judged to have a moderate risk of bias, as they combined quantitative ratings with self-administered questionnaires. Although these included some validated tools, the lack of blinding and potential influence of participant expectations could not be fully excluded (23, 27, 29). Two studies were assessed as having a low risk of bias in this domain. In both cases, usability was measured using well-established and psychometrically validated instruments as SUS administered consistently across participants. Despite the absence of blinding, the objectivity and standardization of the measurement tools substantially reduced the potential for systematic measurement error or subjective distortion (21, 24). Finally, bias in selection of the reported result (D7) was moderate across studies, reflecting the absence of preregistered analysis plans and the potential for selective reporting, particularly in pilot or feasibility designs (21–24, 27, 29). Taken together, the predominance of serious risk of bias limits confidence in the estimated effects. The available evidence should be interpreted as feasibility and the translation of these findings to routine clinical practice should remain cautious until confirmed in adequately studies.

## 4 Discussion

This systematic review examined the usability, acceptability, and preliminary clinical impact of digital rehabilitation interventions in pwMS, focusing on sensor-based technologies, Kinect-based systems. To the best of our knowledge, this is the first systematic review specifically focused on the usability of mobile applications and sensor-based technologies for motor rehabilitation in pwMS. To date, usability has rarely been investigated as a primary outcome in the context of motor rehabilitation. A recent scoping review mapped how usability is addressed across digital health technologies for pwMS, reporting limited use of standardized assessment tools and high methodological heterogeneity, particularly in early-phase developments (10). High usability is not merely a desirable feature; it is a fundamental prerequisite for the successful implementation of digital rehabilitation tools across neurological populations (30–33). Different studies in stroke rehabilitation have demonstrated that virtual reality and robotic systems offer high perceived usability, motivation, and engagement, particularly when interfaces are intuitive, supported by real-time feedback, and tailored to user capabilities. At the same time, cognitive or motor complexity and setup challenges were frequently cited as barriers to adoption, limiting long-term use and adherence (22–24, 26, 27, 29). Similar findings have been reported in Parkinson's disease, where usability has also emerged as a key determinant of adherence and clinical uptake. Reviews of wearable sensor systems and app-based interventions emphasize that technologies designed with user-centred approaches and reduced motor or cognitive demands are more likely to support sustained use and user satisfaction (34–36).

The collected evidence in pwMS demonstrates that these digital solutions generally show good usability, high acceptability, and promising feasibility for motor rehabilitation. In particular, studies employing interactive and personalized interventions, such as exergames using the Kinect platform or mobile applications integrating personalized coaching, goal-setting, and self-monitoring, reported consistently high adherence rates and favourable user experiences, suggesting these elements significantly enhance user engagement (21, 22, 24, 26–28).

Regarding preliminary clinical outcomes, the included studies revealed encouraging results in several functional domains, such as improvements in self-reported physical activity, balance, lower limb strength, and cognitive-motor performance. Specifically, Kinect-based interventions led to measurable enhancements in gait speed, balance control, and coordination tasks, whereas mobile applications primarily improved participant motivation, adherence, and self-reported physical activity (21, 22, 28). Similarly, sensor-based tools like wearable inertial sensors and pressure-sensitive platforms demonstrated potential benefits in balance, vestibular function, and overall motor engagement, although results were variable and sometimes limited by methodological constraints such as small sample sizes and the absence of objective clinical measures (23, 29). Nevertheless, despite good feasibility and user engagement, strong evidence for efficacy and effectiveness of digital technologies in motor rehabilitation for pwMS, regarding objectively measured clinical outcomes is still lacking.

Across the included studies, differences were observed in how usability was reported for mobile applications and sensor-based systems. Mobile apps were generally reported as highly usable, particularly when they incorporated behaviour change techniques such as goal-setting, reminders, and progress tracking, and when their interfaces were designed to be simple and intuitive. These characteristics allowed for autonomous, daily use and integration into personal routines with minimal supervision (24–27).

Sensor-based technologies, including inertial sensors, pressure-sensitive platforms, and Kinect-based systems, also received high usability ratings, especially when paired with real-time visual feedback and adaptive difficulty levels. However, these systems often required more structured environments and sometimes a higher initial cognitive or physical demand. In contrast to mobile apps, which were almost exclusively used in unsupervised, home-based contexts, sensor-based interventions were more frequently delivered in supervised or semi-supervised formats (21–23, 28, 29).

This contrast highlights the importance of tailoring the choice of technology to both the functional profile of the patient and the therapeutic setting. While mobile apps may offer greater flexibility and ease of access, sensor-based tools may provide richer motor feedback and task precision, at the cost of slightly higher setup demands. Ultimately, both modalities can be highly usable if designed with attention to accessibility, simplicity, and clinical integration.

Several studies highlighted specific features that may facilitate the integration of digital rehabilitation technologies into clinical practice. A notable finding was the limited need for prolonged training or familiarization: in most interventions, participants were able to use the digital platforms autonomously after a brief introduction or single supervised session. This was particularly evident in studies employing exergames or mobile applications with intuitive interfaces and real-time visual feedback, suggesting that even individuals with mild-to-moderate disability can engage with such tools effectively and independently (22–24, 27, 29).

Another key element emerging from the review is the active role of the therapist within digital interventions. A user-centred design approach emerged as a key facilitator for intervention success, underscoring the importance of involving patients and clinicians directly in the co-design and iterative refinement of digital rehabilitation technologies. In several protocols, therapists did not serve merely as observers or remote supervisors, but provided meaningful feedback based on system-generated performance data, adjusted task difficulty, and maintained therapeutic interaction through synchronous or asynchronous channels. This hybrid model, combining automated feedback with individualized clinical input, allowed for increased personalization and motivational support, potentially enhancing adherence and clinical outcomes (21, 24, 29). These findings challenge the perception of digital rehabilitation as fully automated or impersonal, and instead support its integration as an interactive and therapist-guided modality within broader care pathways. From a framework perspective, usability should be considered not merely as an implementation factor, but primarily as a prerequisite that may enable engagement and adherence, thereby plausibly contributing to clinical impact through improvements in motor rehabilitation outcomes. Across the included studies, usability and motor outcomes were reported as separate endpoints and none of the interventions tested a direct association between usability scores, adherence metrics and changes in motor performance. This limitation precludes formal evaluation of whether usability or adherence mediates clinical benefit. Nevertheless, interventions that achieved higher usability ratings or positive user experience often also reported improvements in gait, balance or physical activity, together with good adherence to the prescribed sessions. In particular, kinect-based exergaming and virtual rehabilitation systems that were perceived as easy to use, enjoyable and engaging frequently showed gains in gait speed, balance or lower-limb function, while mobile app and sensor-based interventions with favourable usability or acceptability profiles tended to report increased physical activity levels, motivation or self-reported functional improvements. In this sense, future trials should more directly examine the relationship between usability, adherence and motor outcomes by correlating usability metrics or digital engagement data with changes in standardised motor assessment measures. While this link remains to be demonstrated, it is plausible that more usable platforms, particularly when they support sustained adherence to training sessions, may contribute to better motor rehabilitation outcomes in pwMS.

Ensuring high usability is a critical prerequisite for the effectiveness of digital rehabilitation tools in pwMS. Motor impairments and cognitive deficits can interfere with the ability to interact with technology. From this point of view interfaces must be intuitive, accessible, and minimally demanding from both a physical and cognitive standpoint. Poor usability can lead to frustration, decreased engagement, and ultimately low adherence, undermining the potential therapeutic benefits of the intervention. Several studies included in this review reported high usability scores and positive user feedback, often attributed to the implementation of clear visual cues, real-time feedback, and adaptive difficulty levels (22, 23, 27, 29). These features facilitated autonomous use, even in home-based contexts, and supported patient engagement over time. From a clinical perspective, good usability not only enhances the patient experience but also serves as a gateway to behavioural activation and therapeutic adherence. It enables patients to interact with digital systems confidently and consistently, making it more likely that the intervention will be integrated into their daily routines and sustained over time. In this regard, usability should be considered a foundational design criterion rather than a secondary outcome.

Digital technologies offer concrete opportunities to support the behavioural components of motor rehabilitation in clinical practice. Several of the reviewed interventions incorporated key elements of behavioural therapy, including goal setting, self-monitoring, feedback, and motivational reinforcement (25–27). These features align with well-established principles used by clinicians to promote adherence and autonomy in physical activity programs. When integrated into mobile platforms, such components allow patients to track progress, receive structured prompts, and remain engaged in motor tasks outside the clinical setting.

From a clinical perspective, these digital tools can be used to extend therapeutic influence beyond in-person sessions, helping patients to maintain regular motor activity even in the presence of fluctuating symptoms or motivational challenges. For example, therapists can leverage app-based data to provide individualized feedback, adjust goals, or reinforce behavioural achievements, thus creating a continuous therapeutic loop. In this way, mobile applications do not merely deliver content but act as dynamic behavioural supports that can complement therapist-led interventions, encourage long-term adherence, and promote an active lifestyle in pwMS (37, 38).

The evaluation of usability appears particularly relevant in light of emerging treatment scenarios that integrate immersive and interactive technologies. In this context, instruments such as SUS, SUTAQ and MARS could be used to capture users perceptions of functionality, acceptability, and satisfaction, highlighting their role in quantitatively and objectively supporting the design, implementation, and clinical integration of digital rehabilitation tools. Innovative solution like exergames may serve as a valuable complement to conventional rehabilitation, especially when designed to be intrinsically motivatingsuch as through the incorporation of narrative elements or progressive levels of difficulty. A notable example is a protocol for a randomized controlled trial on effectiveness of digital therapeutics in chronic neurological disabilities, that features a virtual dance instructor to promote physical activity (39). Such an approach has the potential to enhance patient engagement and adherence, particularly if the application demonstrates high usability.

Several methodological limitations were identified across the included studies, which constrain the generalizability of the findings. The heterogeneity of age in the included study raises the possibility of overgeneralizing the findings. In particular, although most studies targeted adults with MS, one feasibility study evaluated a mobile-app intervention in adolescents (mean age 16.6 years, SD ± 1.2) with minimal disability (median EDSS 1.5) (26). The inclusion of this study breadens the scope of the review by suggesting that digital rehabilitation tools may also be feasibile in youth with MS; interpretation for this age group must be interpreted with caution, as evidence is currently limited to a single pilot study. Further research specifically designed for adolescent population is needed to clarify age-related differences in usability, adherence and motor outcomes. Most interventions were tested on small samples, frequently in pilot or feasibility designs, limiting the statistical power to detect clinically meaningful changes. Intervention durations were frequently limited to short-term programs, typically ranging from a few sessions to 12 weeks, with no follow-up assessments to evaluate long-term effects or maintenance of treatment benefits. Considerable heterogeneity was observed in the outcome measures used to assess motor performance, adherence, and usability. While some studies employed validated instruments such as the SUS or objective gait measures (e.g., 6MWT, T25FW), others relied on *ad hoc* questionnaires or unstructured feedback, making cross-study comparisons difficult. Adherence metrics varied substantially across interventions; some studies quantified adherence as percentage of prescribed sessions completed, while others as wearable device wear-time, or in other ways, making more complex the interpretation of these findings. The lack of standardized, domain-specific assessment tools hinders the ability to draw firm conclusions about the effectiveness and user experience associated with digital rehabilitation technologies in pwMS. In addition to these design-related limitations, concerns emerged regarding the overall methodological quality of the included studies. Both randomized and non-randomized trials were frequently judged as having “some concerns” or “serious” risk of bias. In randomized trials, these concerns often stemmed from inadequate information on allocation procedures, deviations from intended interventions, and the absence of pre-specified analysis plans. In non-randomized studies, high risk of bias was particularly evident in the domains of confounding, missing data, and deviations from the intended intervention protocol. These methodological shortcomings are especially relevant given that the primary outcome of this review was usability. The absence of validated tools or blinding procedures in several studies may have inflated usability scores through social desirability or participant bias. Furthermore, the reliance on complete-case analyses without appropriate statistical handling of attrition or adherence deviations could have introduced systematic distortions in estimating user experience. These methodological limitations contribute to the overall risk of bias and constrain the interpretability of the findings, particularly regarding their generalizability to real-world clinical contexts. Another limitation of this review is the relatively small number of included studies (*n* = 9), resulting from the rigorous methodological criteria applied. In particular, one highly promising study regarding a telerehabilitation app developed through a user-centred design framework to deliver home-based therapeutic exercise (40), was excluded because its sample involved mixed populations during its initial stage rather than focusing specifically on pwMS, which limited its comparability with the clinical and usability outcomes reported in the other included studies. Although this exclusion reduces the completeness of the overview regarding the spectrum of digital solutions available for motor rehabilitation in MS, the study nonetheless underscores the potential of digital rehabilitation and highlights the importance of user-centered design in developing accessibletools. This further supports the need for additional research in this area.

Despite these methodological limitations, the findings of this review suggest that digital rehabilitation technologies, may serve as valuable adjuncts to conventional rehabilitation programs for pwMS. These tools can support the delivery of personalized motor rehabilitation across diverse contexts, ranging from unsupervised, home-based environments to structured clinical settings. In particular, mobile applications have demonstrated strong potential for promoting autonomy and long-term engagement, especially when designed with adaptive features, self-monitoring, and individualized coaching components. Conversely, sensor-based technologies and Kinect-based systems, while often requiring supervised or semi-supervised environments, have shown high usability and acceptability, and may enhance motor performance through real-time feedback and interactive training paradigms. Across all modalities, the usability of digital tools emerged as a critical factor in facilitating user engagement, adherence, and overall therapeutic feasibility. Interventions developed through user-centred design approaches, incorporating feedback from patients and clinicians, were more likely to yield intuitive interfaces and sustained use. These findings underscore the importance of usability as a prerequisite for clinical integration and as a mediator between technological innovation and behavioural activation. Notably, the interpretability of these results must be tempered by the overall risk of bias identified across the included studies. As usability was the primary outcome of interest, limitations such as the absence of validated instruments, lack of blinding, and inconsistent handling of missing data may have led to overestimations of acceptability or feasibility. Therefore, while digital tools show promising potential to extend therapeutic influence beyond in-person sessions, particularly through hybrid care models, the strength of these conclusions remains contingent upon improvements in methodological rigor and standardization. Future trials should adopt robust designs, include blinded and objective usability assessments where feasible, and report analysis plans transparently to better establish the real-world applicability of these interventions.

To fully harness the clinical potential of digital rehabilitation technologies, future studies should adopt large-scale, methodologically rigorous randomized controlled trials with sufficient statistical power and extended follow-up periods to evaluate the sustainability of effects. Standardization of outcome measures is urgently needed, to enable meaningful cross-study comparisons and meta-analytic synthesis. In parallel, further research should investigate how these tools can be effectively integrated into multidisciplinary care pathways, ensuring alignment with therapeutic objectives, clinical workflows, and individual patient preferences. Addressing these challenges will be essential to move beyond preliminary feasibility and toward the development of evidence-based recommendations for the routine clinical adoption of digital rehabilitation interventions in pwMS.

## Data Availability

The datasets presented in this study can be found in online repositories. The names of the repository/repositories and accession number(s) can be found in the article/Supplementary Material.
