# Supplementary material for: From usability to clinical impact: a systematic review of digital technologies for motor rehabilitation in multiple sclerosis
Source: Front Digit Health. 2026 Feb 16;8:1692875. doi: 10.3389/fdgth.2026.1692875 (PMC12950694; doi:10.3389/fdgth.2026.1692875)
Supplement: Supplementary file 1 [file Supplementaryfile1.docx]

Supplementary Material

# Full search strategy for each database

**PUBMED**: ("Multiple Sclerosis"[Mesh] OR "multiple sclerosis" OR "MS") AND ("smartphone" OR "mobile application" OR "mobile app" OR "tablet" OR "mHealth" OR "mobile health" OR "digital health" OR "digital therapeutics" OR “Serious Games”) AND ("rehabilitation" OR "motor recovery" OR "physical therapy" OR “physical activity” OR "gait" OR "walking" OR "balance" OR "mobility") AND ("adherence" OR "compliance" OR "engagement" OR "feasibility" OR "usability" OR "acceptability" OR "user experience" OR "satisfaction" OR "retention")

**SCOPUS**: TITLE-ABS-KEY("multiple sclerosis" OR MS) AND

TITLE-ABS-KEY("smartphone" OR "mobile application" OR "mobile app" OR "tablet" OR "mhealth" OR "mobile health" OR "digital health" OR "digital therapeutics" OR “Serius Games”) AND

TITLE-ABS-KEY("rehabilitation" OR "motor recovery" OR "physical therapy" OR “physical activity” OR "gait" OR "walking" OR "balance" OR "mobility") AND

TITLE-ABS-KEY("adherence" OR "compliance" OR "engagement" OR "feasibility" OR "usability" OR "acceptability" OR "user experience" OR "satisfaction" OR "retention")

**EMBASE**: ('multiple sclerosis'/exp OR 'multiple sclerosis':ti,ab OR ms:ti,ab) AND

('smartphone'/exp OR smartphone:ti,ab OR 'mobile application':ti,ab OR 'mobile app':ti,ab OR tablet:ti,ab OR 'mhealth':ti,ab OR 'mobile health':ti,ab OR 'digital health':ti,ab OR 'digital therapeutics':ti,ab OR 'serious games':ti,ab) AND

('rehabilitation'/exp OR rehabilitation:ti,ab OR 'motor recovery':ti,ab OR 'physical therapy':ti,ab OR 'physical activity’:ti,ab OR gait:ti,ab OR walking:ti,ab OR balance:ti,ab OR mobility:ti,ab) AND

(adherence:ti,ab OR compliance:ti,ab OR engagement:ti,ab OR feasibility:ti,ab OR usability:ti,ab OR acceptability:ti,ab OR 'user experience':ti,ab OR satisfaction:ti,ab OR retention:ti,ab)

**Web Of Science**: ("Multiple Sclerosis" OR "multiple sclerosis" OR "MS") AND ("smartphone" OR "mobile application" OR "mobile app" OR "tablet" OR "mHealth" OR "mobile health" OR "digital health" OR "digital therapeutics" OR “serious games”) AND ("rehabilitation" OR "motor recovery" OR "physical therapy" OR “physical activity” OR "gait" OR "walking" OR "balance" OR "mobility") AND ("adherence" OR "compliance" OR "engagement" OR "feasibility" OR "usability" OR "acceptability" OR "user experience" OR "satisfaction" OR "retention")
